# Bisretinoid phospholipid and vitamin A aldehyde: shining a light

**DOI:** 10.1194/jlr.TR120000742

**Published:** 2021-02-06

**Authors:** Hye Jin Kim, Janet R. Sparrow

**Affiliations:** 1 Department of Ophthalmology, Columbia University, New York, NY, USA; 2 Department of Pathology and Cell Biology, Columbia University, New York, NY, USA

**Keywords:** autofluorescence, glycerophospholipid, lipid peroxidation, lipofuscin, phosphatidylethanolamine, photodegradation, retina, phospholipase D, phospholipase A_2_, A2-DHP-PE, A2-dihydropyridine-PE, A2-GPE, A2-glycerophosphoethanolamine, all-*trans*-retinal dimer-E, all-*trans*-retinal dimer-ethanolamine, A2PE, phosphatidyl-pyridinium bisretinoid, GPE, glycerophosphoethanolamine, NRPE, *N*-retinylidene-PE, PLA_2_, phospholipase A_2_, PLD, phospholipase D, RDH, NADPH-dependent retinol dehydrogenase, RPE, retinal pigment epithelium, λ_max_, absorbance maximum

## Abstract

Vitamin A aldehyde covalently bound to opsin protein is embedded in a phospholipid-rich membrane that supports photon absorption and phototransduction in photoreceptor cell outer segments. Following absorption of a photon, the 11-*cis*-retinal chromophore of visual pigment in photoreceptor cells isomerizes to all-*trans*-retinal. To maintain photosensitivity 11-*cis*-retinal must be replaced. At the same time, however, all-*trans*-retinal has to be handled so as to prevent nonspecific aldehyde activity. Some molecules of retinaldehyde upon release from opsin are efficiently reduced to retinol. Other molecules are released into the lipid phase of the disc membrane where they form a conjugate [*N*-retinylidene-PE (NRPE)] through a Schiff base linkage with PE. The reversible formation of NRPE serves as a transient sink for retinaldehyde that is intended to return retinaldehyde to the visual cycle. However, if instead of hydrolyzing to PE and retinaldehyde, NRPE reacts with a second molecule of retinaldehyde, a synthetic pathway is initiated that leads to the formation of multiple species of unwanted bisretinoid fluorophores. We report on recently identified members of the bisretinoid family, some of which differ with respect to the acyl chains associated with the glycerol backbone. We discuss processing of the lipid moieties of these fluorophores in lysosomes of retinal pigment epithelial cells, their fluorescence characters, and new findings related to light- and iron-associated oxidation of bisretinoids.

**Figure:**
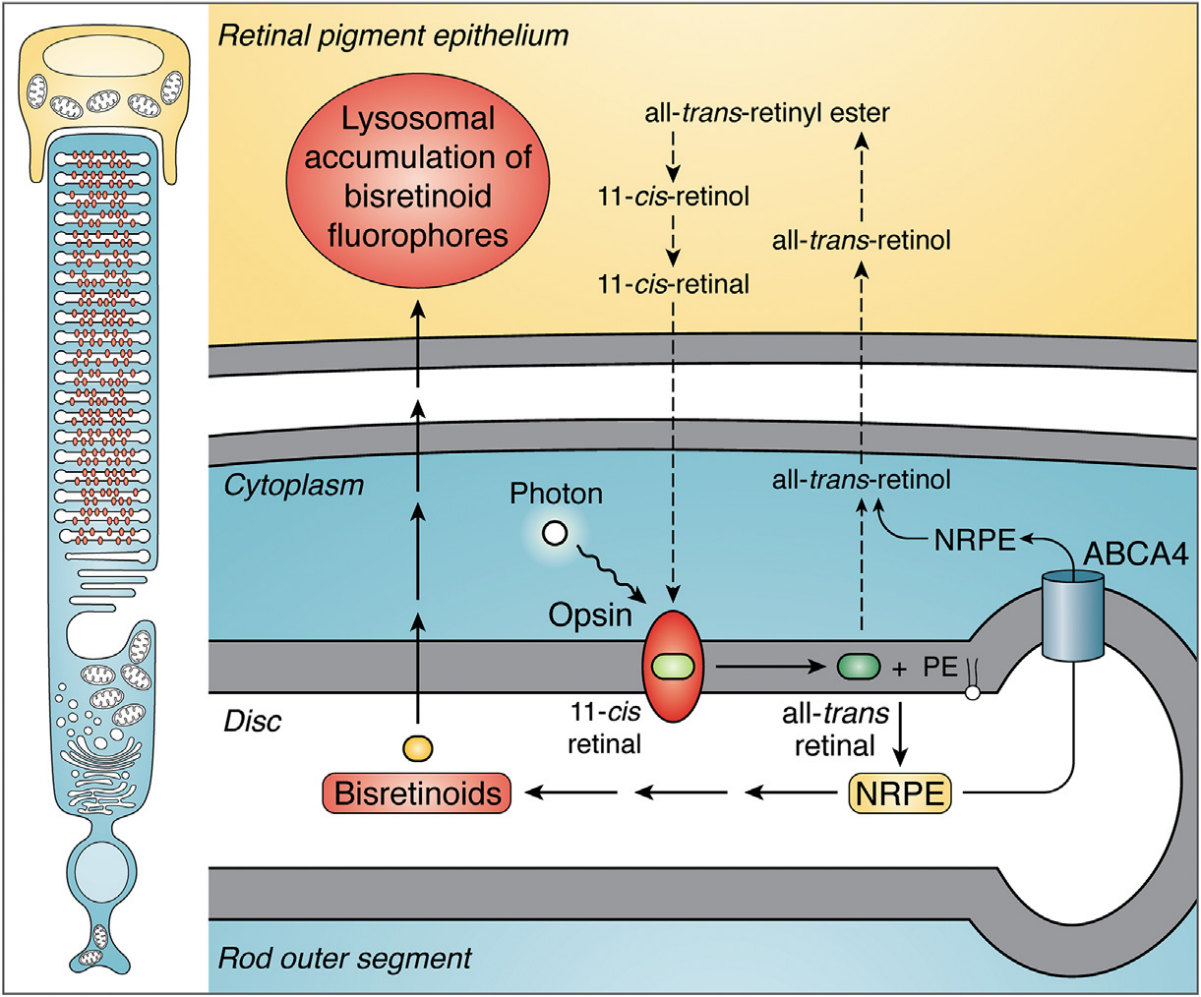

The outer segment compartment of the photoreceptor cell exhibits a distinct lipid and protein composition to support vision. Indeed, more than 90% of the membrane proteins in the rod photoreceptor outer segment disc membrane are rhodopsin (1), and the lipid phase of the photoreceptor outer segment disc membrane is dominated by phospholipids. The ratio of phospholipid to rhodopsin is 100:1 (2). The percent of phospholipid that is PE also exceeds that of other cell membranes (3), and at least two PEs associate with each molecule of rhodopsin. Retinaldehyde that is released by opsin after photon absorption binds to PE and forms *N*-retinylidene-PE (NRPE). The formation of NRPE likely reflects an effort on the part of the photoreceptor cell to protect against acute aldehyde damage and to shepherd vitamin A aldehyde so that it remains within the visual cycle. However, delayed clearance of NRPE paves the way for reaction with a second retinaldehyde and irreversible formation of toxic bisretinoids. In this review, we will update readers on progress made in this field since the related thematic issue of this journal was published in 2010 (4).

## Formation of bisretinoid fluorophores by reaction of vitamin a aldehyde with lipid

**Fig. 1:**
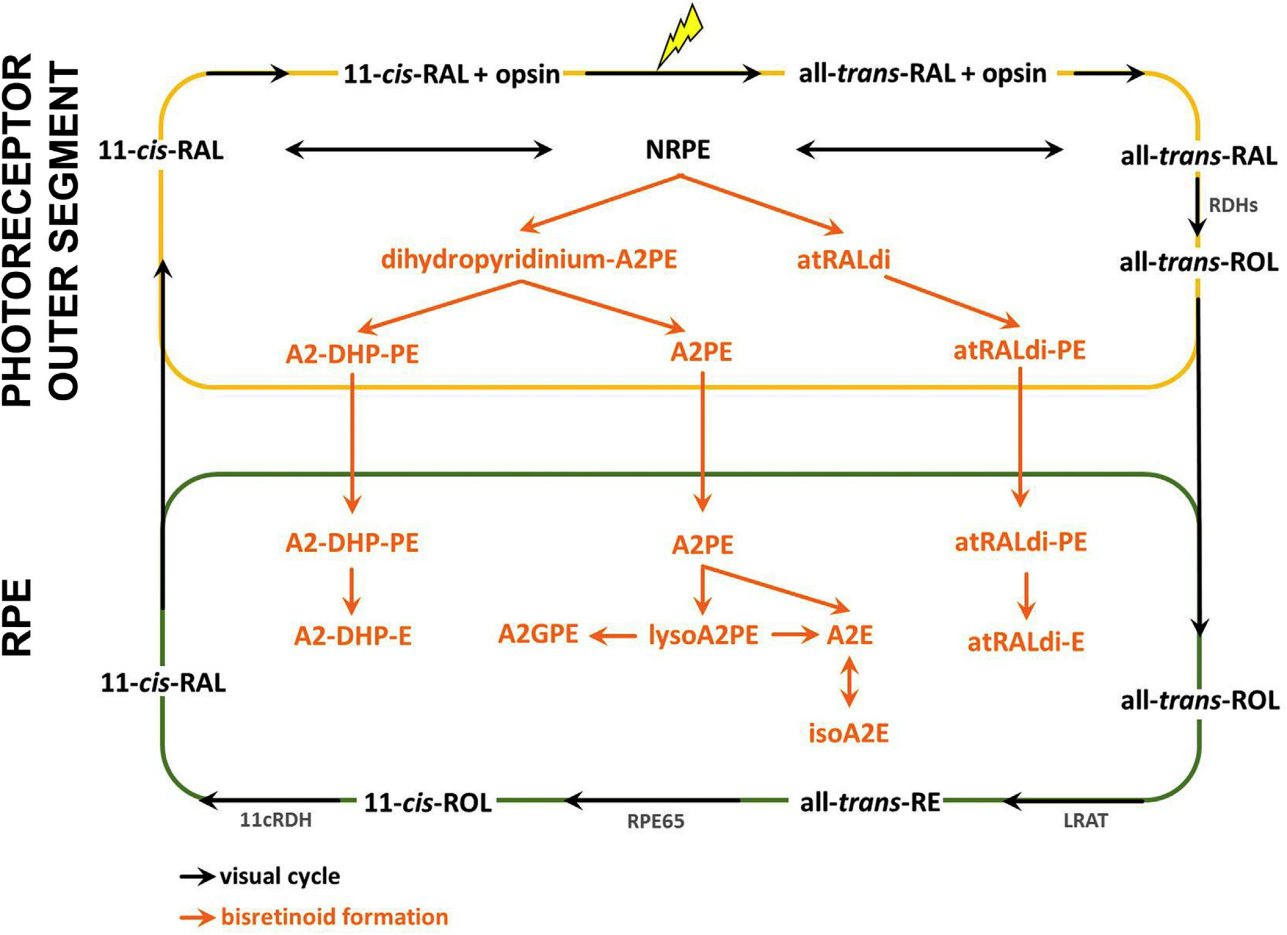
Retinoid cycle of retina. The visual chromophore 11-*cis*-retinal forms a covalent Schiff base with lysine 296 (Lys 296) of opsin. Vision is initiated when a photon is captured by 11-*cis*-retinal; as a result, the chromophore is isomerized to all-*trans*-retinal. With all-*trans*-retinal still covalently bound to opsin, the activated pigment transitions to the metarhodopsin II conformation, the Schiff base is hydrolyzed, and all-*trans*-retinal is reduced to all-*trans*-retinol by RDHs. Alternatively, some all-*trans*-retinal reacts with PE in the lipid bilayer of photoreceptor outer segments to form NRPE, which is transported by ABCA4 and then hydrolyzes to release PE and all-*trans*-retinal. The latter is subsequently reduced to all-*trans*-retinol. Within the RPE cell, all-*trans*-retinol is esterified by the enzyme lecithin retinol acyl transferase (LRAT) and is isomerized from the all-*trans* configuration to the 11-*cis*-retinol by RPE65. The alcohol is then oxidized by 11-*cis*-retinol dehydrogenase (11cRDH) to 11-*cis*-retinal. The bisretinoid synthesis pathway (orange) is initiated when NRPE, rather than hydrolyzing to all-*trans*-retinal and PE, reacts with a second molecule of retinaldehyde. A multi-step pathway leads to formation of the intermediate dihydropyridinium-A2PE. Automatic oxidation of dihydropyridinium-A2PE with loss of two hydrogens generates A2PE, the immediate precursor of A2E and loss of one hydrogen generates A2-DHP-PE; phosphate hydrolysis of the latter produces A2-DHP-E. A2E, lysoA2PE, and A2-GPE are produced from A2PE. Via an alternative pathway, all-*trans*-retinal dimer forms from the condensation of two all-*trans*-retinals. Reaction of the all-*trans*-retinal dimer with PE with formation of a protonated Schiff base linkage generates all-*trans*-retinal dimer-PE (atRALdi-PE), and phosphate hydrolysis of the latter yields all-*trans*-retinal dimer-ethanolamine (atRALdi-E).

After absorption of a photon of light, the 11-*cis*-retinaldehye chromophore of visual pigment isomerizes to all-*trans*-retinaldehyde, thereby initiating a series of conformational rearrangements leading to the phototransduction cascade (Fig. 1). Because it bears a reactive aldehyde, all-*trans*-retinaldehyde must be reduced to the less reactive alcohol (all-*trans*-retinol) by NADPH-dependent retinol dehydrogenases (RDH8, RDH11, and RDH12) in the photoreceptor cell (5, 6, 7, 8, 9, 10). To facilitate this deactivation, some molecules of retinaldehyde react with the primary amine of PE in the outer segment disc membrane, thereby forming the adduct NRPE via a Schiff base linkage (C=C–N) (11). This is a mechanism by which NRPE serves to sequester reactive all-*trans*-retinal. NRPE is also the ligand that binds photoreceptor-specific ABCA4 in outer segments (12, 13, 14, 15, 16, 17, 18). The function of ABCA4 is to transport NRPE across the lipid bilayer to the cytoplasmic face of the disc membrane where NRPE hydrolyzes and all-*trans*-retinaldehyde is released and reduced to all-*trans*-retinol by RDHs (6, 7, 8). The 11-*cis* isomer of NRPE (*N*-11-*cis*-retinylidene-PE) can also be flipped by ABCA4 from the luminal to the cytoplasmic leaflet of disk membranes; this activity is presumed to prevent excess levels of 11-*cis*-retinal (16).

If not efficiently reduced, NRPE also reacts nonenzymatically and irreversibly with a second retinaldehyde molecule to form toxic di-retinal fluorophores (bisretinoids) within the lipid bilayers of the photoreceptor outer segment (Fig. 1). To rid outer segments of these randomly produced light absorbing molecules, the photoreceptor cell undergoes a process of outer segment membrane renewal during which bisretinoids are transferred to retinal pigment epithelium (RPE) and become the lipofuscin of these cells (4, 19, 20, 21). Deficiency in ABCA4 due to gene mutation in humans and mice leads to elevated levels of bisretinoid fluorophores (7, 22, 23, 24).

Incubation of bovine outer segments with all-*trans*-retinal mimics bisretinoid formation in vivo, and by optimizing the detection of these bisretinoid species, their formation by all-*trans*-retinal reactivity was confirmed (25). Indeed, adjusting the ratio of all-*trans*-retinal to PE in synthetic mixtures to determine how the two precursors affect bisretinoid production showed that the yield of bisretinoid saturates when the ratio of equivalents of all-*trans*-retinal to PE (egg-PE as starting material) is 4:2; no further increase occurred with eight equivalents of all-*trans*-retinal (8:2 ratio) (25). Conversely, increasing the concentration of PE from an equivalent ratio of 4:1 through 4:8 (all-*trans*-retinal to PE) was associated with a steady increase in bisretinoid product. These findings indicated that the concentration of PE in the environment of the outer segment membrane has a marked effect on the rate of bisretinoid synthesis. Formation of NRPE, the first step in the synthetic pathway, is likely the most facile reaction. In the *Abca4*^−/−^ mouse, a mouse model exhibiting accelerated bisretinoid formation, the level of PE in the outer segment membrane was reported to be increased (22). It has been suggested that a higher level of PE in *Abca4*^−/−^ mice may facilitate sequestering of all-*trans*-retinal as NRPE, thereby accelerating recovery of the rod photoresponse after a 30–40% bleach (26).

The unsaturated fatty acid 22:6n-3 (DHA) is abundant in the outer segments of photoreceptor cells, and DHA content has been shown to have several effects on retina (27), including susceptibility to light damage. When 22:6n-3-containing PE was used as precursor in the synthetic reaction mixture versus egg-PE (a mixture of fatty acid moieties), phosphatidyl-pyridinium bisretinoid (A2PE) production was found to be greater with the latter. Then again, if free DHA was added to the egg-PE/all-*trans*-retinal reaction mixture, the yield of A2PE was increased. Under deprotonating conditions associated with the addition of base (triethylamine) to the reactants, the generation of A2PE was increased. Altogether, not only is the phospholipid PE the reactant that combines with all-*trans*-retinal to form bisretinoid, the lipid composition of outer segments may also impact the extent to which the reaction may be favored.

## Structural features of bisretinoids

**Fig. 2:**
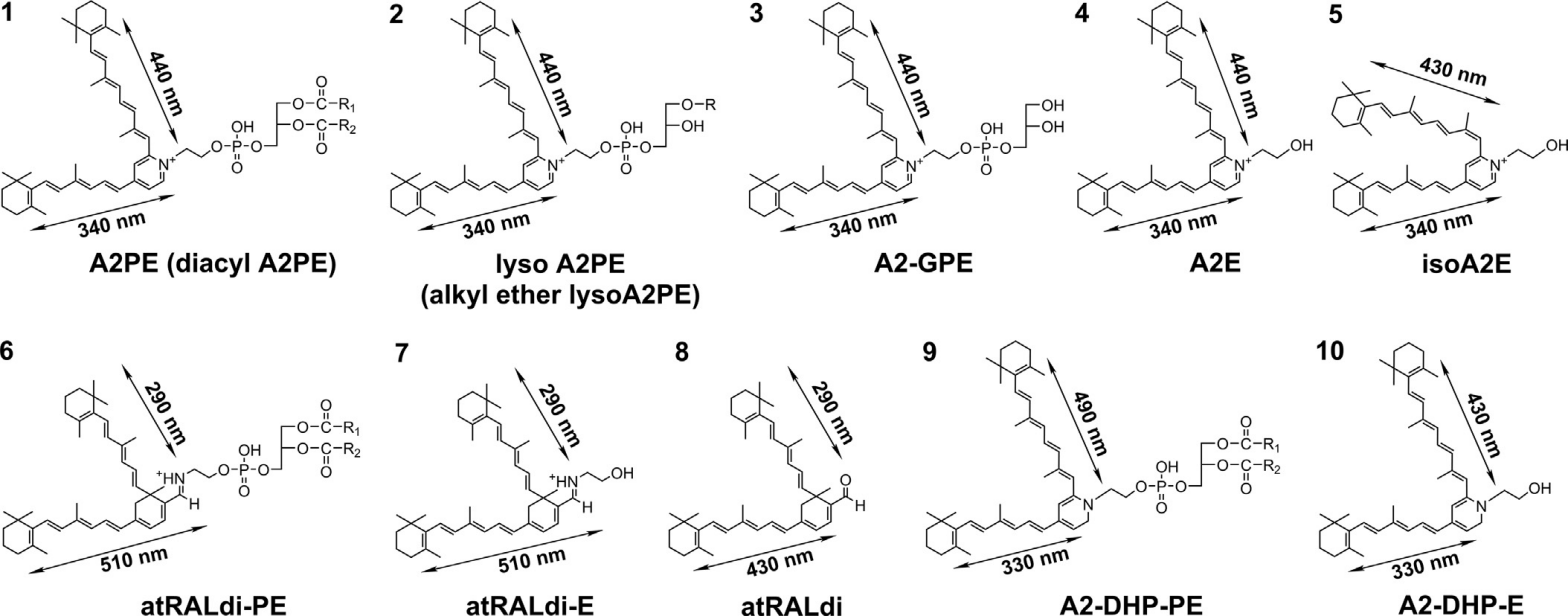
Structures and UV-visible absorbance maxima (shown in nm) of the fluorophores that constitute the bisretinoids of retinal lipofuscin. Absorbance maxima of these bischromophores can be assigned to the shorter and longer side-arms of the molecules. For all-*trans*-retinal dimer-PE and all-*trans*-retinal dimer-E, the absorbance generated from the long-arm exhibits a bathochromic shift (red-shift) due to protonation of the imine functional group (–CH=N–). R, R_1_, and R_2_ are fatty acids with various carbon numbers and multiple double bonds.

Several members of the family of bisretinoids of RPE lipofuscin have been isolated and structurally characterized. All of these fluorophores form by reaction of retinaldehyde in a 2:1 ratio (A2) with glycerol-based phospholipids linked to an ethanolamine by means of a phosphate ester. The bisretinoids of retina consist of a complex mixture of fluorophores that have been identified by chromatography and MS. They have been detected in human and mouse eyes and they have been characterized structurally (25) (Fig. 2). Chromatographic quantitation in murine eyes has revealed an age-related accumulation of each of these known bisretinoids (25, 28, 29, 30). Based on noninvasive quantitative fundus autofluorescence in human subjects, these fluorophores also increase with age in human subjects (31). Bisretinoids are present at elevated levels in mice with a null mutation in the *Abca4* transporter, the gene causative for recessive Stargardt macular degeneration. A signature feature of bisretinoids is that they have two absorbance peaks, one in the UV range and the other in the visible spectrum (Fig. 2). These fluorophores include the pyridinium-containing molecules A2-glycerophosphoethanolamine (A2-GPE) (29), A2E, and isomers of A2E (7, 22, 23, 24, 32, 33, 34, 35, 36, 37); dimers of all-*trans*-retinal having a cyclohexadiene head group (all-*trans*-retinal dimer) (24, 28, 38), the associated protonated Schiff base conjugate (28, 38), and the uncharged A2-dihydropyridine-PE (A2-DHP-PE) (30). Notably, all of these compounds are fluorescent molecules that exhibit absorbances in both the UV and visible range, a signature feature of bisretinoids.

The first bisretinoid to be identified was A2E (32, 33, 34) (Fig. 2, compound 4); A2 in the nomenclature indicates its formation from two vitamin A-aldehyde molecules. The pyridinium bisretinoid (14, 15, 16, 20, 21) structure of A2E (C_42_H_58_ON; molecular weight, 592) was confirmed by extensive nuclear magnetic resonance studies and by total synthesis (32, 33, 34). A2E formation begins in photoreceptor outer segments when all-*trans*-retinal, instead of being reduced to all-*trans*-retinol, reacts nonenzymatically with PE in a 2:1 ratio thus forming the precursor A2PE (Fig. 2, compound 1). A2E is released when A2PE is acted upon by phospholipase D (PLD) in RPE lysosomes. The double bonds along the side arms of A2E are all in the *trans* (*E*) position. In addition, *Z*-isomers of A2E that have double bonds at the C13-C14 (isoA2E) (Fig. 2, compound 5), C9-C10, and C11-C12 positions are also all detectable in human and mouse RPE (28, 34, 39). These pigments exhibit absorbances in both the UV and visible regions of the spectrum [A2E: absorbance maximum (λ_max_), 440 and 340 nm; iso-A2E: λ_max_, 430 and 340 nm]. The polar head of A2E consists of an aromatic ring carrying a permanent positive charge conferred by a quaternary amine nitrogen. The nitrogen does not undergo deprotonation (34, 40), and the charge on the pyridinium nitrogen is neutralized by a counter ion. The aromatic ring also exhibits excellent stability.

Another bisretinoid that is characterized by a central pyridinium ring housing a quaternary amine nitrogen (Fig. 2, compounds 3) exists as an A2-adduct on glycerophosphoethanolamine (GPE) (A2-GPE) (C_45_H_65_O_6_NP; molecular weight, 746) (41). Direct bisretinoid adduct formation on GPE would indicate that in addition to A2-adducts on PE, GPE is accessible for reaction. GPE is the ethanolamine ester of glycerophosphoric acid. In human retina, levels of GPE are 22% of PE (3). The significance of this relatively high content is not known. GPE, a key product of PE catabolism, is generated as a result of phospholipase A_2_ (PLA_2_)-mediated cleavage of the acyl chains at both the *sn*-1 and *sn*-2 position of the glycerol backbone; further hydrolysis can yield ethanolamine and glycerophosphate. Alternatively, GPE could also be produced from plasmalogens (42), a class of glycerophospholipid with a vinyl-ether moiety at the *sn*-1-position of the glycerol backbone; plasmalogens are highly susceptible to oxidation. The bisretinoid A2-DHP-PE has a noncharged dihydropyridine ring at its core (Fig. 2, compound 9); this structure was confirmed by HPLC-ESI-tandem MS with corroboration by Fourier transform infrared spectroscopy and modeling using density functional theory (30). As with the other bisretinoids, this lipofuscin pigment is a fluorescent compound with absorbance maxima at 490 and 330 nm. A2-DHP-PE was identified in human, mouse, and bovine eyes, and we found that A2-DHP-PE forms in reaction mixtures of all-*trans*-retinal and PE. The stability of A2-DHP-PE was evinced by its detection in mouse eyecups and in human and bovine RPE, and with results demonstrating that A2-DHP-PE accumulates with age (30). Another bisretinoid fluorophore of RPE lipofuscin also absorbs in the short-wavelength region of the visible spectrum (17, 18, 23). This pigment, all-*trans*-retinal dimer (λ_max_, 430 and 290 nm) (Fig. 2, compound 8), forms from the condensation of two all-*trans*-retinals and is present in RPE lipofuscin as Schiff base conjugates with either PE or ethanolamine [all-*trans*-retinal dimer-PE and all-*trans*-retinal dimer-ethanolamine (all-*trans*-retinal dimer-E), respectively] or as unconjugated all-*trans*-retinal dimer. The pigments all-*trans*-retinal dimer-PE (Fig. 2, compound 6) and all-*trans*-retinal dimer-E (Fig. 2, compound 7) absorb in the visible range at about 510 nm; the “red” shift relative to all-*trans*-retinal dimer is attributable to protonation of the Schiff base linkage. The protonation state of the Schiff base linkage in all-*trans*-retinal dimer-PE and all-*trans*-retinal dimer-E is pH dependent (28).

The compound all-*trans*-retinal dimer contains a cyclohexadiene ring from which two polyene arms extend: seven double-bond conjugations on the long arm and four on the short arm. All-*trans*-retinal dimer-E and all-*trans*-retinal dimer-PE are dimers of all-*trans*-retinal attached to PE via an imine function group (–C=N) with a protonation state that is pH dependent (38) (Fig. 2, compounds 6 and 7).

The most recently discovered member of the bisretinoid family is alkyl-ether-lysoA2PE (1-alkyl ether-2-lysoA2PE) (1-octadecyl-2-lyso-*sn*-glyceroA2PE) presenting with a single alkyl chain at the *sn*-1 position (Fig. 2, compound 2) (42). This bisretinoid forms by reaction of two vitamin A aldehydes with the ethanolamine head-group of a glycerophospholipid having an ether bond at the *sn*-1 position rather than the more common ester linkage. This structural assignment was based on molecular mass (*m/z* 998), UV-visible absorbance maxima (340 and 440 nm), and a retention time corroborated by biomimetic synthesis using all-*trans*-retinal and GPE analogs as starting materials. UPLC profiles of retinal extracts acquired from human donor eyes revealed that alkyl-ether-lysoA2PE was detectable in RPE but not neural retina. The structure of alkenyl-ether-lysoA2PE, an analog of alkyl-ether-lysoA2PE, including the saturated hydrocarbon long chain and vinyl ether linkage at *sn*-1 of glycerol phosphate moieties, is more complex than A2E. Specifically, due to the negative charge on the glycerol phosphate moiety, alkenyl-ether-lysoA2PE is likely to resist the tight packing required to form micelles with SDS in an aqueous milieu, while A2E with to its cationic polar head group would readily associate with SDS. On the other hand, alkenyl-ether-lysoA2PE may be able to aggregate in association with the hydrophobic retinaldehyde side-arms in aqueous conditions, thus conferring greater photooxidation under blue light radiation.

## Processing of the lipid moiety in lysosomes

Bisretinoid fluorophores are deposited in RPE as a result of two processes: random reactions of vitamin A aldehyde in photoreceptor cell outer segments and phagocytosis of shed photoreceptor outer segment discs by RPE. These fluorophores accumulate with age in the lysosomal compartment of RPE cells and constitute the lipofuscin of retina.

**Fig. 3:**
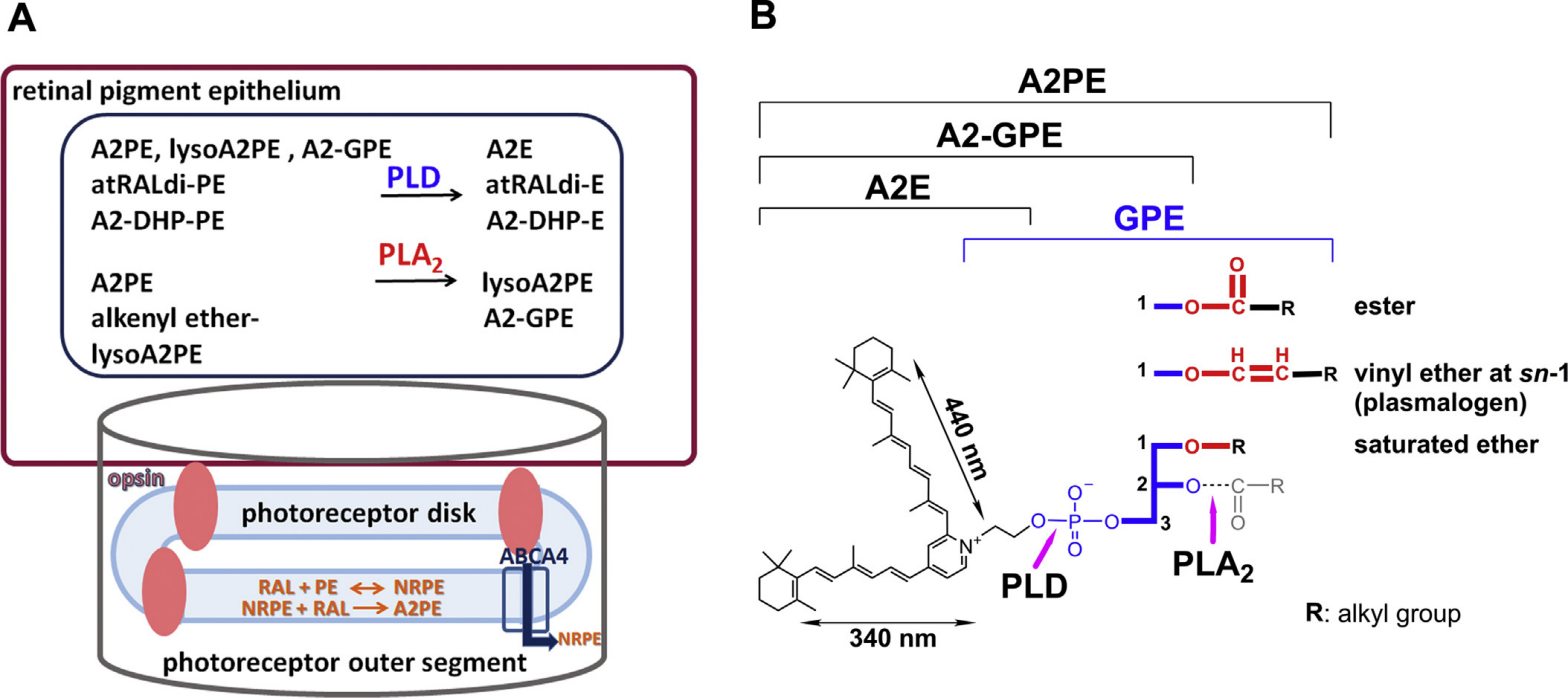
A: Schematic summarizing bisretinoid production in photoreceptor outer segments with processing in RPE. RAL, retinaldehyde. B. Structure of A2PE, A2-GPE, and A2E with UV-visible absorbance (nanometers). The structure includes an ether-linked (–O–) saturated alkyl chain at the *sn*-1 position; a vinyl ether (-O-C=C-) at the *sn*-1 position (plasmalogen) with alkyl groups; and an ester (–O–C=O–) linkage at the *sn*-1 position with alkyl groups. Positions on the GPE that are subject to hydrolysis by PLA_2_ and PLD are indicated (pink arrows). Cleavage by PLA_2_ yields lysoA2PE. Cleavage by PLD yields A2E. Carbon numbers on the glycerol backbone are indicated by 1, 2, and 3. Absorbance peaks at 440 and 340 nm can be assigned to the long and short arms of the molecule, respectively.

**Fig. 4:**
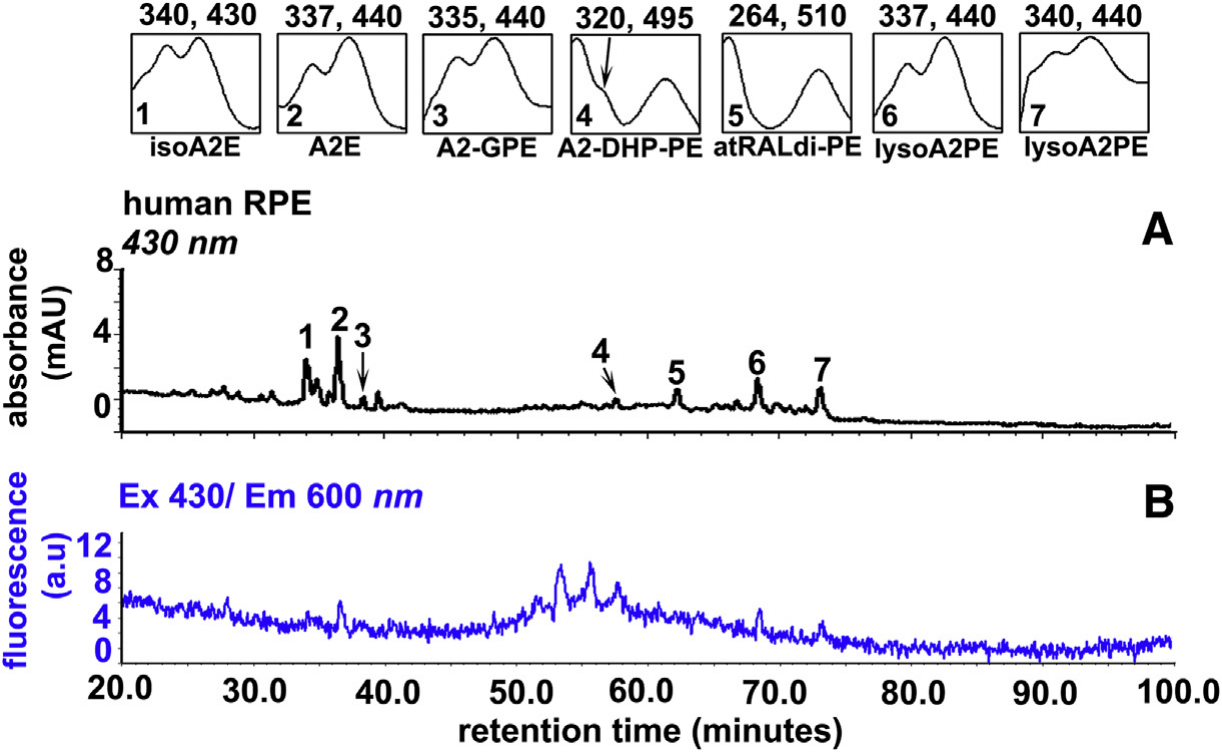
UPLC profile of an extract of human RPE [donor age 74, 1 eye (A, B)]. The chromatogram represents analysis of six eyes. A: Chromatogram with monitoring at 430 nm absorbance. Top insets: UV-visible absorbance spectra of isoA2E, A2E, A2-GPE, A2-DHP-PE, atRALdi-PE, and lysoA2PE species. B: Fluorescence monitoring at an excitation of 430 nm and emission of 600 nm.

**Fig. 5:**
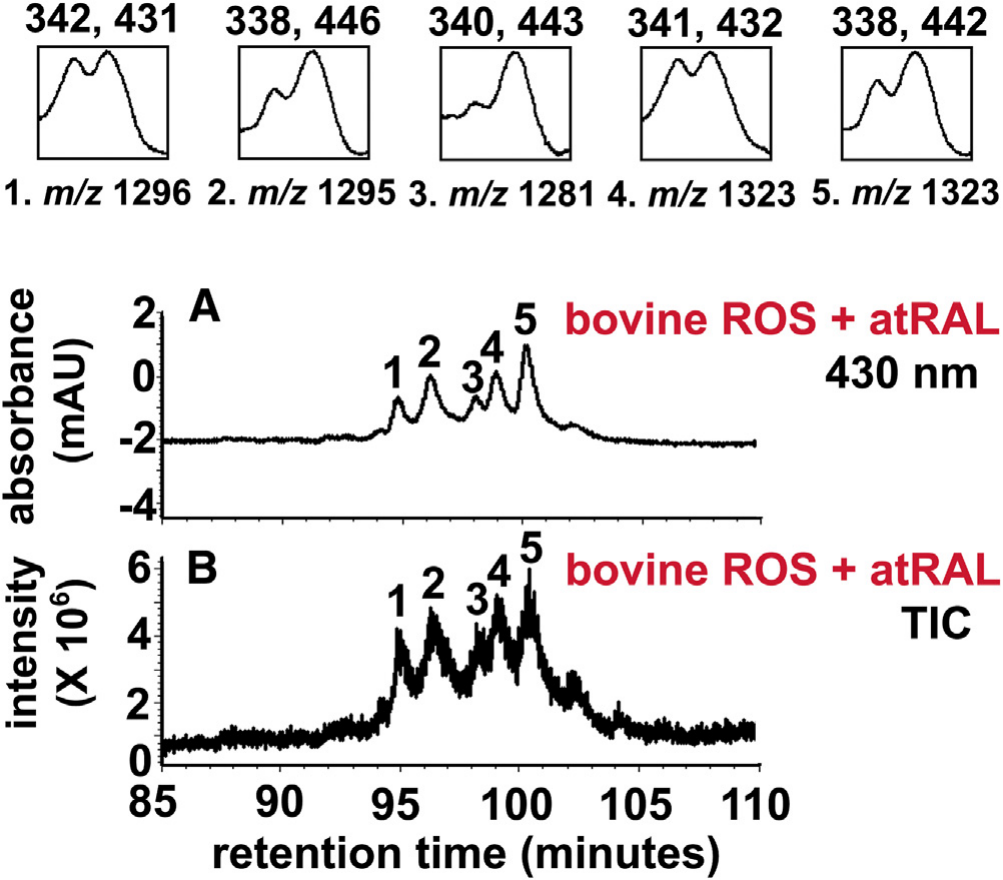
UPLC profiles derived from chloroform extracts of bovine rod outer segments (ROS) (four eyes per sample) incubated with 2 mM atRAL (all-*trans*-retinal) in 2% DMSO in DBPS, in the dark at 37°C for 3 days. Chromatographic separation by UPLC-PDA-MS with monitoring at 430 nm (A) and total ion chromatogram (TIC) (B). Insets: UV-visible absorbance spectra of peaks ∼1–5 from A. Peaks ∼1–5 exhibited corresponded ion at *m/z* 1,296, 1,295, 1,281, 1,323, and 1,323, respectively. A2PE species detected in B exhibit *m/z* 1,323.0 consistent with stearic acid (C18:0) and DHA (C22:6) attached by ester linkages to GPE.

The lysosomal enzyme PLD catalyzes the cleavage of the phosphodiester bond of glycerophospholipids thereby generating phosphatidic acid and a free ethanolamine (Fig. 3) (43, 44, 45). In the case of diacyl A2PE species, PLD-mediated enzymatic hydrolysis (37, 39, 46, 47) releases phosphatidic acid and A2E (pyridinium compound). Evidence for this cleavage event was obtained when A2PE was incubated in the presence of PLD and A2E appeared in chromatographic profiles (46). Hydrolytic activity in lysosomes isolated from liver and RPE also released A2E from A2PE (47). Additionally, this activity was inhibited by the PLD inhibitor calphostin C and by a protease inhibitor cocktail. After phosphate cleavage of A2PE, no further degradation of the molecule occurred (29, 37). The enzymatic processing in lysosomes appeared to be efficient: A2E was always a substantial peak in RPE extracts (Fig. 4), and A2PE was present at relatively low levels (37). In in vitro assays, A2E was also shown to be released from A2-GPE by PLD-mediated activity; however, the abundance of A2-GPE in RPE extracts indicated that this reaction is not favored. Because A2-GPE does not contain fatty acid chains at either the *sn*-1 or the *sn*-2 ester linkage, PLD-mediated hydrolysis at phosphodiester moieties of glycerophospholipid constituents of A2PE is evidently not affected by fatty acid chains attached to the glycerol backbone (Fig. 3). On the other hand, the relative abundance of A2-DHP-PE in mouse eyecups and human and bovine RPE indicated that A2-DHP-PE is more refractory to cleavage. Consequently, this bisretinoid retains the phospholipid-derived tail. Another bisretinoid in RPE that retains the phospholipid moiety is all-*trans*-retinal dimer-PE. A2PE, A2-DHP-PE, and all-*trans*-retinal dimer-PE are not single molecular species. Rather, each consists of a series of bisretinoids having fatty acids of varying lengths and numbers of double bonds, for instance 22:6 (DHA) and 18:0 (stearic acid) attached at the *sn*-1 and *sn*-2 through ester or ether linkages (Fig. 5).

Recently, we have added to our understanding of enzyme processing by showing that PLA_2_ can also process A2PE (1-alkyl ether-2-acyl-A2PE) (i.e., an ether bond at the *sn*-1 site and an ester bond at the *sn*-2 site) by mediating cleavage at the ester bond positioned at the *sn*-2 site so as to produce lysoA2PE presenting with a single alkyl chain at the *sn*-1 position (42) (Fig. 3). The detection of the bisretinoid alkyl-ether-lysoA2PE not in neural retina but in human RPE (Fig. 4) indicated that the PLA_2_ activity likely resides in RPE. In addition, we showed that the plasmalogen-lysoA2PE can subsequently undergo hydrolysis under acidic conditions expected for RPE lysosomes. This process yielded A2-GPE and thereby disclosed a new mechanism contributing to the final processing of bisretinoid (Fig. 3).

## Why do bisretinoids accumulate in the lysosomal compartment of rpe cells?

RPE lipofuscin differs from other forms of lipofuscin. Specifically, the bisretinoid composition of RPE lipofuscin distinguishes this material from lipofuscin forms that consist of cross-linked oxidatively modified proteins (48) or protein accumulations (i.e., subunit c of mitochondrial ATP synthase), as in neuronal ceroid lipofuscinosis, a lysosomal storage disease (49). Other reported molecular constituents of RPE lipofuscin are adducts of 2-(ω-carboxyethyl)pyrrole (50), 4-hydroxynonenal, and malondialdehyde (51) that are derived from oxidative fragmentation of lipid. The oxidative processes that produce these 2-(ω-carboxyethyl)pyrrole-protein adducts could occur in photoreceptor cells before RPE phagocytosis of outer segment membrane. It is just as likely that these products of lipid oxidation are generated within the lysosomal bodies in which RPE lipofuscin is stored; there they would be generated by bisretinoid-initiated photooxidation. Otherwise the spectral properties of the blue-green emitting fluorescent products of lipid oxidation are markedly different (excitation maxima ∼350 nm, emission maxima ∼435 nm) (52) than spectra generated from RPE lipofuscin (53, 54). It has been suggested that bisretinoids such as A2E are amassed in lysosomes of RPE cells because they become trapped after protonation in the acidic environment of the lysosome. It is also assumed that the resulting alkalinization of the lysosome milieu inhibits lysosomal enzymes (55, 56). However, A2E is a quaternary pyridinium salt that does not deprotonate or reprotonate; the positive charge on the pyridinium nitrogen is neutralized by a counterion, probably chloride (25, 26, 43). The bisretinoid A2-DHP-PE presents with an uncharged dihydropyridine ring at its core; it does not undergo protonation and deprotonation (30). Similarly, the bisretinoid, all-*trans*-retinal dimer also accumulates in lysosomal storage bodies; this fluorophore presents with a noncharged cyclohexadiene ring (28, 38).

All-*trans*-retinal dimer can also form a conjugate with PE (all-*trans*-retinal dimer-PE) via a Schiff base linkage that exhibits pH-dependent protonation, and as with unprotonated unconjugated all-*trans*-retinal dimer, all *trans*-retinal dimer-PE accumulates in RPE lysosomes. Moreover, as we have shown here, the final step in the formation of A2E, A2-GPE, and lysoA2PE, all of which are amassed in RPE lysosomes, depends on the activity of at least two hydrolytic enzymes in lysosomes, PLD and PLA_2_ (Fig. 3). Considered together, these findings indicate that one cannot attribute bisretinoid accumulation to inhibition of lysosomal enzyme activity. Reduced activity of lysosomal degradative enzymes, if it were to occur, would result in a generalized increase in protein/peptide accumulation as in lysosomal storage diseases (57), but this is not observed (50). Specifically, a proteomic study of purified lipofuscin granules revealed that the amino acid content was only 2% (w/w) (50). Other investigators have noted (53, 54, 58) that the presence of photoreceptor proteins in preparations enriched in lipofuscin-containing lysosomal organelles (59, 60) is attributable to contamination with unprocessed phagosomes. And finally, the view that RPE lipofuscin accumulates because of inhibition of lysosomal enzymes, cannot be reconciled with the accumulation of this material in all healthy eyes even at young ages (31). Instead, it is likely that RPE lysosomal enzymes that would otherwise degrade the bisretinoid, do not recognize the structures that constitute this material. PLA_2_ may be of additional interest. Toxic sodium iodate, when internalized by ARPE-19 cells, induces increased expression of calcium independent PLA_2_ (61), while benign flecks retina is associated with the gene encoding PLA_2_ group V (62, 63).

## Fluorescence of bisretinoids

Bisretinoids exhibit a central six-membered ring from which extends dual polyene arms terminating in β-ionine rings (Fig. 2). Each of the arms constitutes a distinct system of double bond conjugations with each arm serving as a retinaldehyde-derived chromophore, one arm absorbing in the UV range and the other in the visible region of the spectrum (25, 64). The wavelengths at which bisretinoids absorb are determined by the lengths of the systems of alternating double and single bonds, including the double bonds in the β-ionone and central rings. Accordingly, the UV absorbance can be assigned to the short-arm while the long-arm generates the absorbance in the visible range. Absorbances are in the visible spectrum range from 430 to 510 nm. The phospholipid moiety, if retained, does not make a contribution to an absorbance above 250 nm.

Although the excitation maxima of the various bisretinoid fluorophores varies, their fluorescence emission maxima are similar and generally peak between ∼600 and 620 nm. The retina exhibits an intrinsic autofluorescence that is excited by short-wavelength visible light and that has been monitored in human subjects by in vivo spectrofluorometry (65) and by fluorescence adaptive optics ophthalmoscopy (66), and that in human subjects is imaged clinically by noninvasive confocal scanning laser ophthalmoscopy (67, 68, 69). This autofluorescence has a broad excitation spectrum that peaks between 490 and 510 nm. The fluorescence emission is also broad and centered at approximately 600 nm (69, 70). The spectral characteristics of short-wavelength fundus autofluorescence are consistent with those of RPE lipofuscin (53, 54, 71, 72) and principally with an origin from the bisretinoid fluorescent pigments that are the constituents (64). Moreover, the emission spectra recorded from whole lipofuscin, bisretinoid, and short-wavelength fundus autofluorescence all exhibit red-shifts when excited by progressively longer wavelengths (64).

A significant increase in fluorescence emission is observed with limited photooxidation of A2E and all-*trans*-retinal dimer (discussed below) as compared with the parent compounds (73). Moreover, this increase in fluorescence emission can occur without a change in visible-spectrum absorbance when the oxidation occurs on the short arm of the molecules (73). With further oxidation on the long-arms, fluorescence bleaching is observed.

## Photooxidation and photodegradation of bisretinoid lipofuscin

**Fig. 6:**
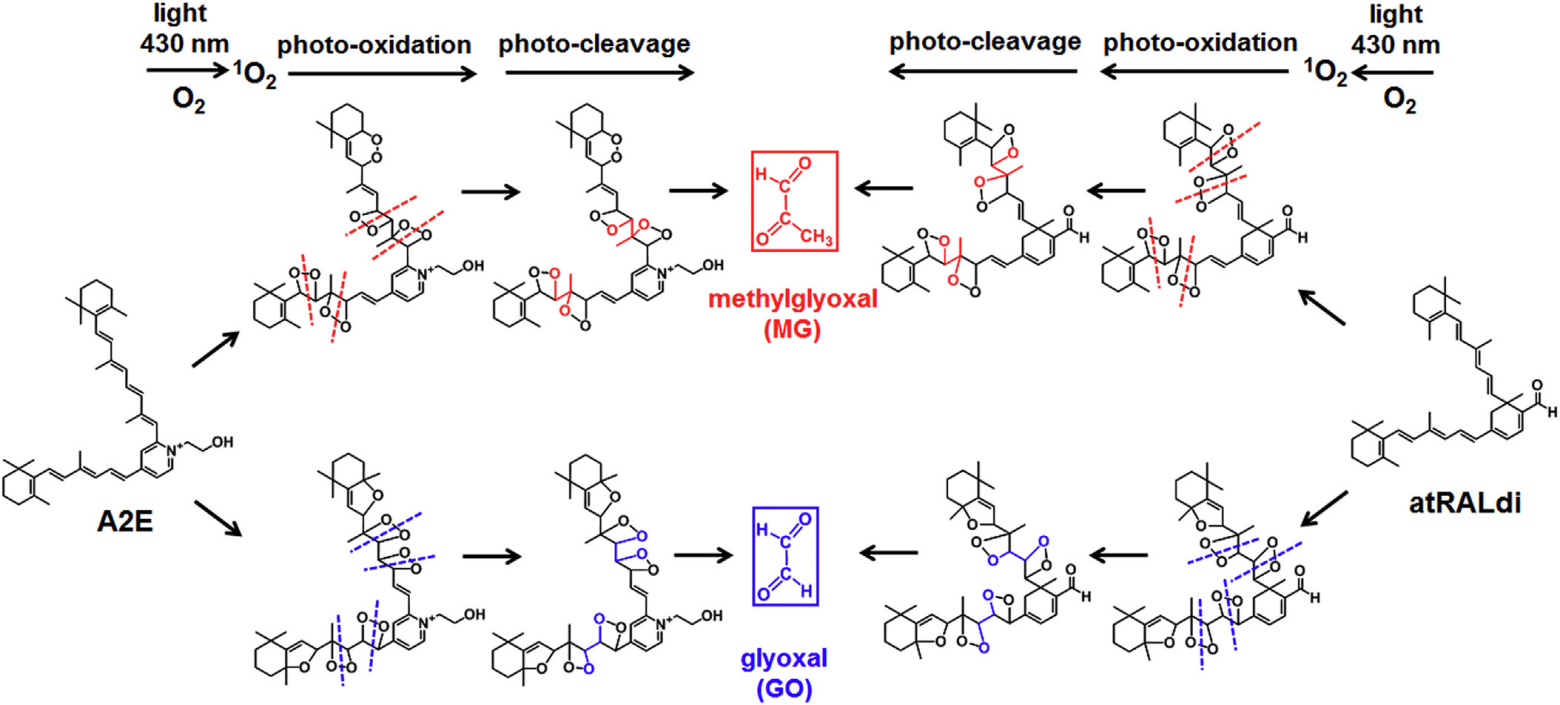
The dicarbonyls methylglyoxal and glyoxal are released from the photodegradation of RPE bisretinoids. Shown here is the photodegradation of A2E and all-*trans*-retinal dimer (atRALdi). Potential cleavage sites at carbon-carbon double bonds are exhibited (dashed lines). Each molecule of A2E and atRALdi could release methylglyoxal or glyoxal depending on the photodegradation patterns. Adapted from (25).

Because the cellular build-up of bisretinoid has adverse consequences for RPE cells, efforts have been made to understand the processes by which these fluorophores are damaging. While the bisretinoids of lipofuscin are unlikely to undergo lysosomal digestion, these fluorophores are nevertheless subject to photodegradation. Accordingly, whole lipofuscin mixtures (74, 75) and individual fluorophores such as A2-GPE (29), A2E (46), and all-*trans*-retinal dimer (28) have been shown to initiate photosensitization reactions that generate superoxide anion and singlet oxygen (28, 46, 76). Singlet oxygen, as a highly reactive form of oxygen, adds to conjugated double bonds along the side-arms of the bisretinoid molecules. In the case of A2E, this oxidation is evidenced in ESI/FAB-MS as mass increments of 16 (molecular weight of oxygen) starting from the molecular ion [M^+^] at *m/z* 592 peak attributable to A2E (46). Oxidized bisretinoids (e.g., mono- and bis-peroxy forms of A2E and those of all-*trans*-retinal dimer) have been detected in human and mouse retina (28, 76). At these unstable oxygen-containing moieties, bisretinoids cleave and release a complex mixture of aldehyde-containing fragments and the dicarbonyls methylglyoxal and glyoxal (Fig. 6) (77, 78, 79) that ravage cellular and extracellular molecules and reflect a link between RPE bisretinoid lipofuscin and the formation of sub-RPE deposits. The photodegradation products released from bisretinoids cross-link protein and suppress matrix metalloproteinase activity (80) and inhibit the proteasome (81). Given that photooxidation of A2E and all-*trans*-retinal dimer has also been shown to incite complement activation (82, 83) together with evidence that these carbonyl-modified proteins are present in deposits (drusen) that accumulate on the basal side of RPE cells in vivo (84, 85), the photodegradation of bisretinoids is likely linked to AMD. These processes likely also contribute to Bruch’s membrane thickening observed in *Abca4*^−/−^ mice (86).

In studies of albino versus pigmented mice and mice raised in continuous darkness versus cyclic light, levels of bisretinoid were found to be lower in mice exposed to higher levels of intraocular light (albino mice and cyclic-light reared mice). Moreover, in mice treated with the antioxidant vitamin E, photodegradation of bisretinoid was suppressed resulting in increased bisretinoid and sparing of photoreceptor cells. Vitamin E intercepts bisretinoid oxidation by scavenging reactive oxygen species generated by bisretinoid photosensitization (77). And finally, these photoreactive processes explain photoreceptor cell degeneration in *Abca4*^−/−^ mice (87, 88) and are a cause of the increased vulnerability of albino *Abca4*^−/−^ mice to retinal light damage (89).

Intracellular iron can also promote the oxidation and degradation of bisretinoid by generating hydroxyl free radical via the Fenton reaction. Specifically, in mice treated with the iron chelator deferiprone, bisretinoid levels, measured by HPLC and noninvasively by quantitative fundus autofluorescence, were elevated (90). Loss of photoreceptor cell viability that is detected by outer nuclear layer thinning was also mitigated in deferiprone-treated albino *Abca4*^−/−^ mice. Conversely, mice exhibiting increased iron in RPE cells due to deficiency in the iron export proteins hephaestin and ceruloplasmin, presented with reduced bisretinoid levels.

## Cones and rod photoreceptor cells

Two types of photoreceptor cells populate the retina. Rod photoreceptors provide vision at low light levels and three types of cones enable color vision and vision in ambient lighting. Common to all of these light-sensitive cells is the visual chromophore 11-*cis*-retinal that binds to opsin molecules specific to each type of photoreceptor. Photon absorption triggers the isomerization of 11-*cis*-retinal to all-*trans*-retinal; continued light perception requires reconversion of the chromophore to the 11-*cis* isomer. The canonical visual cycle operating in RPE cells is responsible for regeneration and provision of the visual chromophore 11-*cis*-retinal to rods and cones in order to sustain photosensitivity. A supply of vitamin A is stored in RPE as esterified fatty acids. Subsequently the energy required to drive the double bond isomerization is derived from the hydrolysis of fatty acid retinyl esters. However, abundant evidence indicates that the efficiency with which the RPE visual cycle generates 11-*cis*-retinal is not sufficient to support cone-mediated vision in daylight. Various additional pathways have been suggested that include: light-dependent reverse isomerization of all-*trans*-retinal bound in the form of NRPE (91); light-dependent pathways involving retinal G protein-coupled receptor (RGR) with all-*trans*-retinal as substrate (92); or light-independent processes that harness the retinoid isomerase isomerase sphingolipid delta(4)-desaturase 1 (DES1) (93, 94). An intraretinal cone-specific visual cycle would likely involve Muller glial cells (95).

Questions have arisen as to whether the high visual chromophore demands of cone photoreceptor cells confer greater susceptibility to bisretinoid formation. While bisretinoid fluorophores are the source of short-wavelength fundus autofluorescence, the signal from foveal and perifoveal cones in humans is attenuated by macular pigment and by the increased optical density of melanin. Also complicating this issue is the possibility of greater photocleavage and loss of bisretinoid in central cone-rich retina due to higher light exposures (96). Thus, the question of cone versus rod bisretinoid formation cannot be addressed by noninvasive quantitative fundus autofluorescence imaging (97) in human subjects.

Nevertheless, some insight was provided by studies of mice deficient in the Nrl transcription factor (Nrl^−/−^) (98). In these mice, rods are largely replaced by cone-like photoreceptor cells. These cells express cone proteins and exhibit cone electrophysiological and structural features. With placement of Nrl^−/−^ mice on a background of *Abca4*^−/−^, bisretinoid levels, as expected, were higher in *Abca4*^−/−^ than in *Abca4*^+/+^ mice. In addition, analysis by HPLC revealed that the cone-dominant eye generated more bisretinoid than the rod-dominant eye. Indeed, when bisretinoid levels were normalized to 11-*cis*-retinal quantity, bisretinoid (per eye) was found to be 6.8-fold higher in cone dominant *Abca4*^−/−^/Nrl^−/−^ mice than in the primarily rod retina in *Abca4*^−/−^ mice.

## Bisretinoids and retinal disease

The efficiency with which the retinoid cycle replenishes the 11-*cis* chromophore of cone and rod visual pigment determines all-*trans*-retinal flux and thus is tightly coupled to the formation of lipofuscin bisretinoids (23, 99, 100, 101, 102). On the other hand, conditions that interfere with clearance of all-*trans*-retinal from the interior of outer segment discs result in accelerated formation of the bisretinoids. For instance, as a consequence of deficient ABCA4-facilitated removal of all-*trans*-retinal from the interior of outer segment discs (12, 15, 17, 103), RPE lipofuscin is elevated in ABCA4-related disease and in *Abca4*^−/−^ mice (22, 23, 104, 105, 106). Mutations in *ABCA4* can present clinically as recessive Stargardt macular degeneration (107), as a form of cone-rod dystrophy, or as retinitis pigmentosa (108). Knockout of the photoreceptor cell enzymes (all-*trans*-retinol dehydrogenases) responsible for detoxifying all-*trans*-retinal (by conversion to all-*trans*-retinol) also leads to enhanced formation of bisretinoid (8, 102). Dysfunctional photoreceptor cells may also generate bisretinoid at accelerated levels. For instance, in acute zonal occult outer retinopathy, short-wavelength autofluorescence is elevated at the border between diseased and nondiseased retina and, at this lesion border, SD-OCT imaging reveals a loss of photoreceptor cell integrity (109). The hyperautofluorescent ring that marks the junction between functional and nonfunctional retina in many cases of retinitis pigmentosa, also signals accelerated bisretinoid formation (110). And finally, in mice homozygous for a targeted deletion of the Mer receptor tyrosine kinase gene (*mer^kd^*; *Mertk*^−/−^), it has been shown that the autofluorescence in the photoreceptor cell debris that accumulates in the subretinal space originates from bisretinoids that form in abundance in this model (111). Moreover, these photoreactive compounds are linked to the mechanisms by which light potentiates photoreceptor cell degeneration in these mice.

## Concluding remarks

PE in the lipid bilayer of photoreceptor outer segments participates in the sequestration and reduction of vitamin A aldehyde by forming a reversible Schiff base linkage (NRPE) with this reactive molecule. The formation of NRPE likely serves to both retain vitamin A within the visual cycle and limit the reactivity of this acutely toxic aldehyde. Under some conditions, NRPE reacts irreversibly with a second molecule of vitamin A aldehyde, thus forming bisretinoid fluorophore. RPE phagocytosis serves to transfer bisretinoid-burdened outer segment discs to the RPE but as has been demonstrated in mice deficient in the receptor tyrosine kinase Mer (111), phagocytosis is not required for the formation of these fluorophores. When known isomers (e.g., *cis* isomers of A2E) and photooxidized forms of bisretinoids are included together with biosynthetic intermediates such as A2PE (Fig. 2, compound 1) and dihydropyridinium-A2PE, at least 27 bisretinoid fluorophores can be identified chromatographically and by MS. The accumulation of bisretinoid by RPE is well-known to have adverse consequences for the cells and is implicated in disease processes.

## Conflict of interest

The authors declare that they have no conflicts of interest with the contents of this article.
